# Taxol alleviates collagen-induced arthritis in mice by inhibiting the formation of microvessels

**DOI:** 10.1007/s10067-017-3646-1

**Published:** 2017-04-28

**Authors:** Juan Xu, Zhitao Feng, Shixian Chen, Junqing Zhu, Xianghui Wu, Xiaoguang Chen, Juan Li

**Affiliations:** 1 Department of Internal Medicine of Traditional Chinese Medicine, College of Traditional Chinese Medicine, Southern Medical University, Guangzhou, Guangdong 510515 China; 2 Department of Traditional Chinese Medicine, Medical College of China Three Gorges University, Yichang, Hubei 443002 China; 3 Department of Rheumatology, Nanfang Hospital, Southern Medical University, No. 1838, North of Guangzhou Avenue, Guangzhou, Guangdong 510515 China; 4 Laboratory Animal Center, Nanfang Hospital, Southern Medical University, Guangzhou, Guangdong 510515 China; 5 Department of Pathogen Biology, School of Public Health, Southern Medical University, Guangzhou, Guangdong 510515 China

**Keywords:** Angiogenesis, Hypoxia-inducible factor-1α, Rheumatoid arthritis, Taxol, Vascular endothelial growth factor

## Abstract

The objective of the present study is to evaluate the inhibitory effects of taxol (PTX) on angiogenesis in a collagen-induced arthritis (CIA) mouse model. Collagen II (C II) and complete Freund’s adjuvant (CFA) were used in C57BL/6 (H-2b) mice to generate the CIA model. Random grouping was performed in the normal control group, CIA model group, PTX 1.5 mg/kg group, PTX 1.0 mg/kg group, and PTX 0.5 mg/kg group. Arthritis index scores, tissue pathology scores, and synovium microvessel density (MVD) analysis were performed. Immunohistochemistry and enzyme-linked immunosorbent assay were used to detect the expression of vascular endothelial growth factor (VEGF) and hypoxia-inducible factor-α (HIF-1α). The correlation between MVD and pathological scores and between MVD and the expression of VEGF as well as HIF-1α in the synovium were also evaluated. After PTX treatment, the three intervention group arthritis index scores were reduced when compared with the CIA group. The total histological scores in the three PTX treatment groups were lower than those in the CIA group. Similarly, PTX significantly alleviated the scores for synovitis, pannus formation, and bone destruction. Compared with the CIA group, the MVD of the three intervention groups decreased in a dose-dependent manner. The expression of VEGF and HIF-1α in synovial tissues and serum also significantly decreased after PTX treatment. Further analysis showed that MVD and pathological scores and MVD and expression of VEGF as well as HIF-1α in the synovium were positively correlated. PTX may alleviate CIA by suppressing angiogenesis, providing new insights into the treatment of rheumatoid arthritis (RA). VEGF and HIF-1α may be targets for PTX suppression of microvessel formation.

## Introduction

Rheumatoid arthritis (RA) is a common chronic inflammatory autoimmune disease that is characterized by persistent synovitis and angiogenesis, resulting in synovial hyperplasia and progressive destruction of bone and cartilage [1–3]. Although the etiology of RA has not been entirely identified, 50% of the risk for the disease has been ascribed to genetic factors and the remaining to the environment and chance [4].

Angiogenesis is a critical feature in RA [5]. A vast body of research has indicated that angiogenesis is involved in the pathogenesis of RA [6]. It is also involved in several physiological events such as inflammation, embryonic organ development, tissue repair, reproduction, and wound healing [7]. In RA, uncontrolled neovascularization can induce synovial hyperplasia and progressive bone destruction by fostering the infiltration of inflammatory cells into the joints, which promotes the process of RA [8]. Consequently, inhibiting the formation of blood vessels may be an effective therapy for RA [9].

The collagen-induced arthritis (CIA) mouse model is one of the most extensively used autoimmune models of RA. It is widely employed to explore potential pathogenic mechanisms and to predict the clinical efficacy and safety of many new RA drugs [10]. The CIA model possesses several pathological features resembling RA, including cartilage degradation, synovial hyperplasia, angiogenesis, and mononuclear cell infiltration. It displays the shortest duration between immunization and disease manifestation. Above all, this kind of mouse model is popular in studies focusing on RA angiogenesis [11].

Taxol (PTX) is a natural complex diterpene alkaloid extracted from the dried inner bark of *Taxus brevifolia*. It primarily interferes with the structure of the inner part of the cell membrane [12]. Nearly 50 years ago, the National Cancer Institute proposed that PTX possesses anti-cancer properties, and since then, PTX has been widely studied as an anti-cancer agent. It is one of the most efficient anti-cancer medicines and has been used to treat various cancer types, especially metastatic breast cancer, non-small-cell lung cancer, ovarian cancer, and Kaposi’s sarcoma [13]. However, recent studies have shown that low-dose PTX can be used to treat inflammation [14], renal [15] and hepatic fibrosis [16], skin disorders [17], axon regeneration [18], and coronary artery restenosis [19], which are non-cancer human diseases. Additionally, the ultra-low non-cytotoxic concentration of PTX has been demonstrated to have immunomodulatory properties. Particularly, several studies have demonstrated that low-dose PTX has anti-angiogenesis effects and that it can depress the product of vascular endothelial growth factor (VEGF) [20] and hypoxia-inducible factor-α (HIF-α) [21] in cancer.

Although many drugs possess anti-inflammatory functions [22], angiogenesis inhibitors focusing on neovascularization in RA are still lacking. Therefore, this research topic merits exploration of potential drugs targeting angiogenesis in RA. This study was designed to evaluate the inhibitory effects of PTX on angiogenesis in CIA models.

## Materials and methods

### Animals

C57BL/6 (H-2b) 6–8-week-old male mice were purchased from the Laboratory Animal Centre of Guangdong province (Guangzhou, Guangdong, China. No. 00102710). The animals were raised in the SPF animal laboratory of Southern Medical University (Guangzhou, Guangdong, China. No. 44007200015700).

### Arthritis induction

CIA induction was performed according to a previous protocol [10]. Chicken type II collagen (CII) was solubilized in 0.1 M acetic acid to a concentration of 2 mg/ml and then emulsified in equal volumes of complete Freund’s adjuvant (CFA). The emulsion (0.1 ml) was injected intradermally at the base of the tail on day 0. On day 14 after the primary immunization, the mice received a booster immunization of 0.1 ml C II in incomplete Freund’s adjuvant (IFA). The C II, CFA, and IFA were purchased from Sigma-Aldrich, St. Louis, MO, USA.

### Experimental group design and treatment

A total of 50 mice were used to induce the CIA mouse model, and the arthritis incidence was 62%. Twenty-four of the mice with obvious arthritis syndrome were randomly divided into four groups: CIA model group, PTX 1.5 mg/kg group, PTX 1.0 mg/kg group, and PTX 0.5 mg/kg group. Each group consisted of six animals. Another six normal mice were considered the normal control group. PTX (Sigma-Aldrich, St. Louis, MO, USA) was solubilized in a 1:1 dilution of ethanol and cremophor EL (Sigma-Aldrich, St. Louis, MO, USA). Normal saline was then added to achieve a final concentration of 4.8 mg/ml PTX in 5% ethanol (weight/volume) and 5% (*w*/*v*) cremophor prior to intraperitoneal injection. All treatment protocols were initiated on day 35 of arthritis onset and continued for the duration of the 14-day study (a total of 8 treatments). PTX was intraperitoneally injected in the treatment groups every other day. The three treatment groups received dosages of 1.5 mg/kg, 1.0 mg/kg, and 0.5 mg/kg. The normal control group and CIA model group received 0.1 ml normal saline on alternate days. All animals were sacrificed by CO_2_ inhalation on day 50 after the last treatment.

### Arthritis assessment

Arthritis assessments were performed by two independent, blinded observers. Both the incidence and the severity of arthritis were evaluated. From the primary immunization (day 0) to the last treatment time point (day 50), the arthritis score was recorded daily. Scoring for each paw was based on an integer from 0 to 4 based on standardized levels of erythema, swelling, and rigidity of the joint as follows: 0: normal, no erythema or swelling; 1: erythema and mild swelling confined to the tarsals or ankle joint; 2: erythema and swelling extending from the ankle to the tarsals; 3: erythema and moderate swelling extending from the ankle to the metatarsal joint; and 4: erythema and severe swelling encompassing the ankle, foot, and digits or ankylosis of the limb. The sum of the scores obtained for four legs represented the arthritis index for each animal, with a maximum possible score of 16 per mouse.

### Histopathological assessment

Hind limbs, including the paws and ankles, were dissected on day 50, and the tissues were immediately placed in 4% paraformaldehyde for 24 h and then decalcified in 10% EDTA for 2 months at 4 °C and embedded in paraffin. Tissue sections (4 μm thick) were stained with hematoxylin and eosin (HE). The stained sections were randomly collected from six mice in each group and evaluated by two trained independent observers who were blinded to the treatment protocols. At least three fields per section were evaluated. The sections were analyzed using a Nikon Eclipse 80i microscope (Nikons, Badhoevedorp, Netherlands) at 200-fold magnification. Histopathologic changes were assessed and scored for three parameters according to a previously reported method [23, 24].

The first parameter was synovitis judged by the thickness of the synovial membrane. Synovitis was scored as follows: 0: less than 3 cells thick; 1: 3–5 cells thick; 2: 6–10 cells thick; 3: 10–20 cells thick; 4: 20–30 cells thick; and 5: beyond 30 cells thick. The second parameter was pannus formation. Pannus formation was quantified as follows: 0: no pannus formation; 1: microvillus present; 2: clear pannus attachment; 3: marked pannus attachment; 4: joint space filled by pannus; and 5: extensive pannus proliferation. The last parameter was bone destruction, which was scored as follows: 0: no erosion visible; 1: minor indentation in the area of capsular attachment; 2: clear erosions of cartilage; 3: erosion extending into the subchondral bone; 4: major erosion of bone and cartilage; and 5: loss of visible cartilage and major bone loss.

### Immunohistochemistry analysis

The 4% paraformaldehyde-fixed limbs were decalcified and paraffin-embedded using standard techniques. Hind limb sections were subjected to immunohistochemical analysis using specific antibodies. The primary antibodies were mouse anti-CD31 antibody (1:100 dilution), mouse anti-VEGF antibody (1:200 dilution), or mouse anti-HIF-1α antibody (1:200 dilution). Horseradish peroxidase-labeled goat anti-rabbit IgG (1:200 dilution) was used as the secondary antibody. All experimental procedures were performed according to the manufacturer’s instructions. These antibodies were purchased from Google Biological Technology, Wuhan, China. The stained samples were randomly selected from each group, and every section was assessed using three typical fields for electron microscopy (×200) to ensure that the visual field covered as much tissue as possible. The expression of CD31 was detected by immunohistochemistry to measure the microvessel density (MVD) in the joint sections. The number of microvascularized tissue areas in the images was counted, and then, the MVD was calculated for each group. The expression of VEGF and HIF-1α in the synovium was analyzed semi-quantitatively using Image-Pro Plus 6.0 to evaluate the integrated optical density (IOD) of every sample. Two operators collected the results independently.

### Enzyme-linked immunosorbent assay analysis

Blood was collected from animals by retroorbital puncture on day 50. Blood samples were centrifuged for 10 min at 3000 rpm after incubation at room temperature for 30 min, and serum was obtained from each sample. The levels of VEGF and HIF-1α in the sera were quantified by enzyme-linked immunosorbent assay (ELISA) according to the manufacturer’s instructions (Cusabio, Wuhan, China).

### Statistical analysis

All values were expressed as the mean ± SD and analyzed using the SPSS statistical package (version 20.0, Armonk, New York, the USA). One-way ANOVA was used to compare group means, and differences between the groups were tested for statistical significance using the least significant difference under the assumption of equal variances, while Dunnett’s T3 was used if equal variances were not assumed. Spearman correlation analysis was performed to evaluate the correlation between MVD and pathological scores and between MVD and the expression of VEGF as well as HIF-1α in the synovium. The reported *p* values are two-tailed, and *p* < 0.05 was considered statistically significant.

## Results

### Effect of PTX on the clinical manifestation and arthritis index of CIA mice

**Fig. 1:**
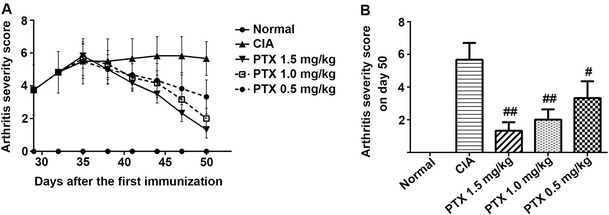
The effect of PTX on the arthritis severity score in CIA mice. **a** Mice were injected with PTX (1.5, 1.0, 0.5 mg/kg) or normal saline eight times from day 35 after the first immunization. The arthritis severity score was recorded. **b** On day 50, the arthritis scores of three intervention groups declined compared with the CIA group. Additionally, the arthritis severity scores in the three PTX treatment groups and the CIA group were higher than that in the normal control group. #*p* < 0.05, ##*p* < 0.01 compared with CIA

No significant differences in arthritis scores were observed between the three PTX treatment groups and the CIA group before the PTX intervention. The arthritis scores decreased in a dose-dependent manner following treatment with PTX (Fig. 1a). On day 50 after the last treatment, the arthritis severity score in the CIA group was higher than that in the normal control group. In the three intervention groups, the arthritis index scores (1.33 ± 0.52, 2.00 ± 0.63, 3.33 ± 1.03) were decreased compared with the CIA group (5.67 ± 1.03; *p* < 0.001, *p* < 0.001, *p* = 0.016). The PTX treatment group arthritis index scores were higher than those in the normal control group (Fig. 1b).

### Effect of PTX on histopathologic scores

**Fig. 2:**
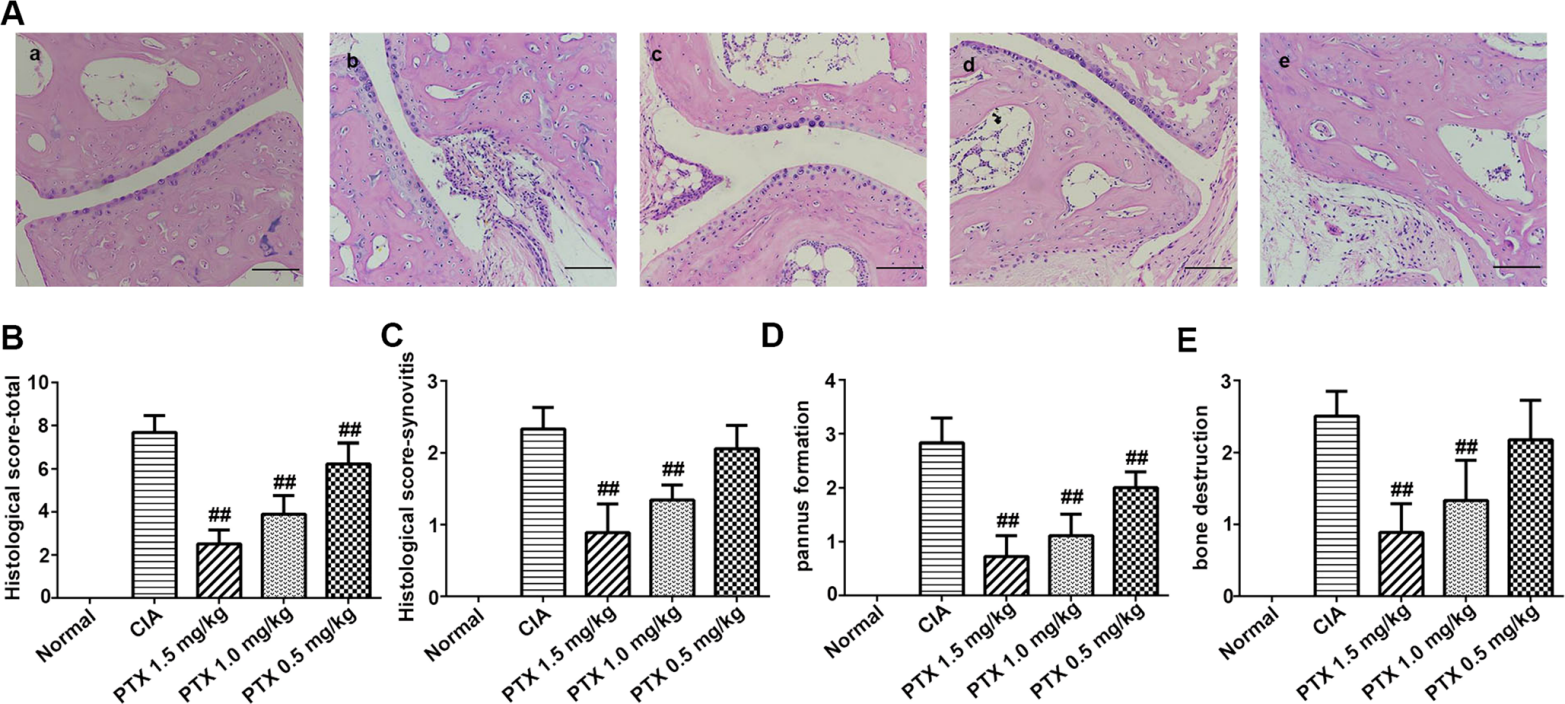
Histological analysis of inflamed joints in CIA mice. **a** The histological analysis was performed using mouse hind paw sections stained with HE (*scale bars* 100 μm): *a* normal group, *b* CIA group, *c* PTX 1.5 mg/kg group, *d* PTX 1.0 mg/kg group, and *e* PTX 0.5 mg/kg group. **b**–**e** Mean ± SEM histological arthritis scores (synovitis, pannus formation, bone destruction, and total) in the normal control group, CIA group, and three PTX treatment groups. #*p* < 0.05, ##*p* < 0.01 compared with the CIA group

Intraperitoneal administration of PTX for CIA had a beneficial effect on the terminal histopathology of CIA. The typical CIA control group showed notable synovitis and pannus formation, as well as erosions extending through the hyaline cartilage and spreading into the subchondral bones. In contrast, the pathological features of the PTX treatment groups differed from the CIA group and the normal group (Fig. 2a). The pannus and synovitis were less extensive, bone erosion was attenuated, or erosion was confined to the marginal area. The total histological scores in the three PTX treatment groups (2.50 ± 0.66, 3.89 ± 0.86, 6.22 ± 0.98) were lower than those in the CIA group (7.67 ± 0.79; *p* < 0.001, *p* < 0.001, *p* = 0.007) (Fig. 2b). Moreover, PTX significantly alleviated the scores for synovitis, pannus formation, and bone destruction. The synovitis scores in the PTX 1.5 mg/kg (0.89 ± 0.40) and 1.0 mg/kg group (1.34 ± 0.21) were less than that in the CIA group (2.33 ± 0.30; *p* < 0.001, <0.001), but the score for the 0.5 mg/kg PTX group (2.06 ± 0.32, *p* = 0.144) was similar to those in the CIA group (Fig. 2c). The scores for pannus formation in the PTX treatment groups (0.72 ± 0.39, 1.11 ± 0.40, 2.00 ± 0.30) were decreased when compared with the CIA group (2.83 ± 0.46; *p* < 0.001, *p* < 0.001, *p* = 0.001) (Fig. 2d). The bone destruction scores in the PTX 1.5 mg/kg (0.89 ± 0.40) and 1.0 mg/kg group (1.33 ± 0.56) were lower than that in the CIA group (2.50 ± 0.35; *p* < 0.001, *p* < 0.001), while there was no difference between the PTX 0.5 mg/kg group and the CIA group in bone destruction scores (*p* = 0.239) (Fig. 2e). Furthermore, the total histological, synovitis, pannus formation, and bone destruction scores in the CIA and three PTX treatment group were higher than those in the normal group.

### Effect of PTX on the synovium MVD

**Fig. 3:**
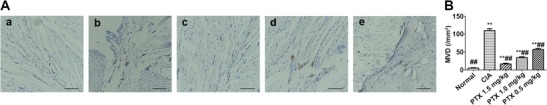
CD31 antibody was used to identify synovium microvessels. **a** Immunohistochemical staining of CD31 in hind paw specimens from each group: *a* normal group, *b* CIA group, *c* PTX 1.5 mg/kg group, *d* PTX 1.0 mg/kg group, and *e* PTX 0.5 mg/kg group. *Scale bars* = 100 μm. **b** The stained samples were assessed by randomly selecting six high-power microscopic fields (×200) for each paw, and images of the representative sections were obtained. The capillary density expressed by the number of CD31+ cells per square field. The immunostained sections were analyzed semi-quantitatively using Image-Pro Plus 6.0. MVD and the results are presented as the mean ± SEM. *p < 0.05, **p < 0.01 compared with the normal control group, #*p* < 0.05, ##*p* < 0.01 compared with the CIA group

We used CD31 antibody to identify synovium microvessels (Fig. 3a). The statisticaldata showed that the average MVD in the normal control group was 5.20 ± 1.60/mm^2^, whereas the values for the CIA and three intervention groups, respectively, were 110.32 ± 5.06, 17.05 ± 1.97, 34.73 ± 2.36, and 57.55 ± 2.72/mm^2^. When compared with the CIA group, the MVD in the three intervention groups decreased in dose-dependent manner (*p* < 0.001, respectively). In contrast, the others groups had a higher MVD than the normal group (*p* < 0.001, respectively) (Fig. 3b).

### Effect of PTX on the expression of VEGF and HIF-1α in the CIA synovium

**Fig. 4:**
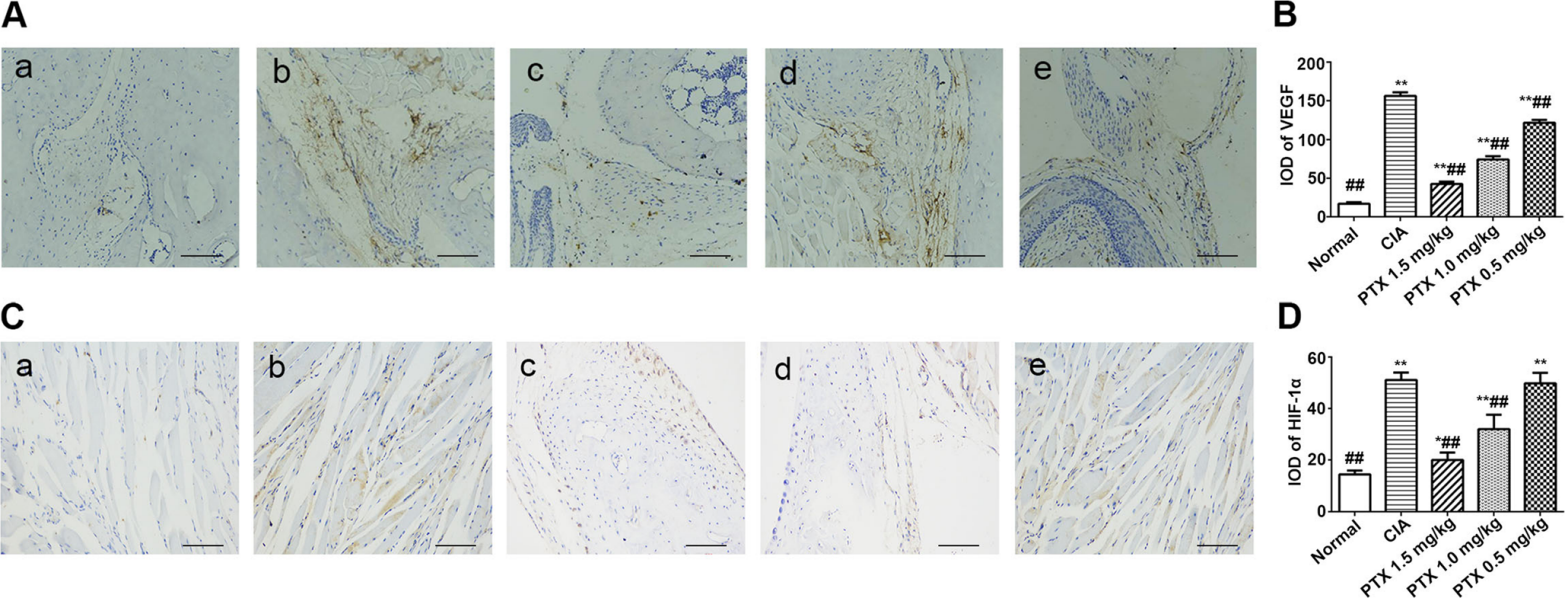
**a**, **c** Immunohistochemical staining of VEGF and HIF-1α in hind paw sections from mice from CIA, normal, and three PTX treatment groups (*scale bar* = 100 μm): *a* normal group, *b* CIA group, *c* PTX 1.5 mg/kg group, *d* PTX 1.0 mg/kg group, and *e* PTX 0.5 mg/kg group. Strong positive staining was observed in the CIA mice, while the mice treated with PTX exhibited weaker staining. **b**, **d** The stained samples were randomly selected in three different microscopic fields (×200) for each paw. Quantification of VEGF and HIF-1α-positive staining using Image-Pro Plus 6.0. Values are presented as the mean ± SEM. *p < 0.05, **p < 0.01 compared with the normal control group, #*p* < 0.05, ##*p* < 0.01 compared with the CIA group

Considering that PTX could suppress angiogenesis in the mouse paw, we next evaluated the expression of the angiogenesis regulators VEGF and HIF-1α (Fig. 4a, c). Strong positive staining was observed in the CIA mice. The results indicated that the levels of VEGF in synovial tissue from the PTX treatment groups and CIA group were higher than those from normal mice (*p* < 0.001, respectively). The level of HIF-1α in the synovium exhibited a similar difference (*p* = 0.015, *p* < 0.001, *p* < 0.001, *p* < 0.001). PTX reduced the expression of VEGF in the three PTX treatment groups (42.38 ± 3.22, 74.30 ± 4.14, 121.69 ± 3.81) compared with the CIA group (156.22 ± 4.75; *p* < 0.001, *p* < 0.001, *p* < 0.001). The level of HIF-1α in the PTX 1.5 and 1.0 mg/kg groups (19.93 ± 2.92, 31.99 ± 5.60) was also weakened when compared with the CIA group (51.10 ± 2.86; *p* < 0.001, *p* < 0.001). Nevertheless, there was no difference in HIF-1α expression in the synovium between the PTX 0.5 mg/kg group and the CIA group (49.72 ± 4.17 vs 51.10 ± 2.86, *p* = 0.521) (Fig. 4b, d).

### Effect of PTX on the expression of VEGF and HIF-1α in peripheral serum

**Fig. 5:**
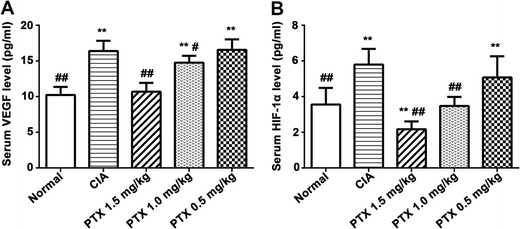
Effect of PTX on the expression of VEGF and HIF-1α in peripheral serum. The levels of VEGF (**a**) and HIF-1α (**b**) in serum were measured by ELISA. Values are expressed as the mean ± SEM. *p < 0.05, **p < 0.01 compared with the normal control group, #*p* < 0.05, ##*p* < 0.01 compared with the CIA group

We further explored the effect of PTX on the level of VEGF and HIF-1α in peripheral serum. The ELISA results showed that serum VEGF and HIF-1α expression levels in the CIA group were significantly higher than those in the normal group (16.40 ± 1.43 vs 10.21 ± 1.13 pg/ml, *p* < 0.001; 5.79 ± 0.89 vs 3.56 ± 0.93 pg/ml, *p* < 0.001). The serum VEGF levels in the three treatment groups were 10.70 ± 1.21, 14.75 ± 0.96, and 16.55 ± 1.47 pg/ml, and the serum HIF-1α levels were 2.17 ± 0.43, 3.47 ± 0.51, and 5.07 ± 1.19 pg/ml. When compared with the CIA group, the serum VEGF and HIF-1α levels were lower in the PTX 1.5 mg/kg (*p* < 0.001, *p* < 0.001) and PTX 1.0 mg/kg groups (*p* < 0.001, *p* = 0.032), and there were no significant differences between the PTX 0.5 mg/kg group and the CIA group (*p* = 0.841, *p* = 0.143). In addition, the serum VEGF levels in the PTX 1.5 mg/kg group were approximately equal to the normal group (*p* = 0.504). Notably, the serum level of HIF-1α in the PTX 1.5 mg/kg group was lower than that in the normal group (*p* = 0.008) and that in the PTX 1.0 mg/kg group was similar to that in the normal group (*p* = 0.860) (Fig. 5a, b).

### Correlation between MVD and pathological scores and between MVD and the expression of VEGF as well as HIF-1α in the synovium

**Fig. 6:**
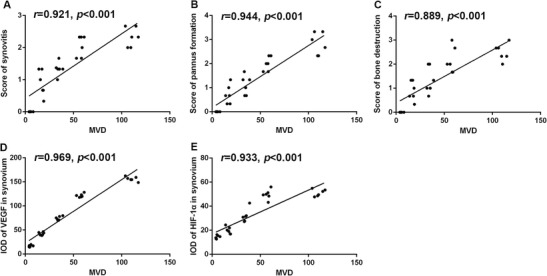
Evaluation of the correlation between MVD and pathological scores and between MVD and the expression of VEGF as well as HIF-1α in the synovium. A positive correlation was found between MVD and the scores for synovitis (**a**), the score for pannus formation (**b**), the score for bone destruction (**c**), and the levels of VEGF (**d**) and HIF-1α (**e**) in the synovium

The synovitis scores were positively correlated to MVD (*r* = 0.921, *p* < 0.001) (Fig. 6a). Similarly, the scores for pannus formation and bone destruction were positively correlated to MVD (*r* = 0.944, *p* < 0.001; *r* = 0.889, *p* < 0.001) (Fig. 6b, c). Moreover, the levels of VEGF, HIF-1α in the synovium, and MVD were also positively correlated (*r* = 0.969, *p* < 0.001; *r* = 0.933, *p* < 0.001) (Fig. 6d, e).

## Discussion

The results of the present study show that PTX treatment can reduce the CIA arthritis score, as evidenced by the reduction of erythema and swelling, in agreement with a precious study. Additionally, the MVD, histological scores, and levels of VEGF and HIF-1α in the synovium and serum were also reduced after PTX treatment in a dose-dependent manner. These findings suggest that PTX may ameliorate CIA by suppressing angiogenesis by targeting VEGF and HIF-1α.

As a broad-spectrum anti-cancer chemotherapeutic agent, PTX can contribute to the inhibition of spindle microtubule dynamics and chromosome segregation [25], thus hampering metaphase-anaphase transitions and ultimately suppressing mitosis and inducing apoptosis in cancer cells [26]. PTX can inhibit the cell cycle in the G0/G1 and G2/M phases and kill proliferating cancer cells by stabilizing the microtubule polymer and suppressing microtubule disassembly [27, 28]. Additionally, previous research has indicated that PTX can suppress cell proliferation and has anti-angiogenic effects. Elegant studies have revealed that PTX has immunoregulatory functions. It can promote the maturation and function of human and murine dendritic cells (DCs) in vitro [29, 30]. Additionally, it can downregulate p38 MAPK signaling in myeloid-derived suppressor cells (MDSCs), leading to fewer MDSCs and diminished production of chronic inflammatory mediators in vivo [31]. In addition, PTX has been reported to attenuate the expression of tumor necrosis factor α (TNF-α) and thrombin-induced solute permeability [32, 33]. Studies have shown that PTX can ameliorate the vascular leak and inflammation in a lipopolysaccharide-induced acute lung injury mouse model. The first study to examine PTX intervention in animal models of RA demonstrated that it can preclude the development of CIA and suppress established clinical disease, radiographic erosions, and anti-CII IgG antibody expression in serum [34]. A. L. Arsenault et al. confirmed that PTX treatment could induce neovascular and synoviocyte component reversion to a naïve synovium morphology [35]. Additionally, PTX has been shown to cause cell arrest at G2/M phase and apoptosis in fibroblast-like synovial cells (FLSs) from RA patients [36]. The current study demonstrated that PTX treatment alleviated CIA arthritis scores, as evidenced by the reduction of erythema and swelling. PTX significantly improved histopathological features based on the decreased scores for synovitis, pannus formation, and bone destruction. This finding is in agreement with the results of previous studies.

Angiogenesis is the development of new capillaries from preexisting vessels, and it plays an indispensable role in the progression of RA [5]. MVD is a critical parameter for angiogenesis. The new blood vessels can transport a large number of inflammatory cells to inflamed sites and provide oxygen and nutrients to the proliferating inflamed tissue to maintain the chronic inflammatory state [9, 37]. The pro-inflammatory cytokines excreted from synovial tissue layer lining cells can trigger RA angiogenesis. During stimulation by inflammatory factors, RA fibroblasts and synovial tissue macrophages produce multiple pro-inflammatory cytokines. Pro-inflammatory cytokines can regulate the expression of growth factors, matrix metalloproteinases (MMPs), adhesion molecules, and chemokines, all of which are essential for the development of angiogenesis [7, 38]. Additionally, an excess of pro-angiogenic factors can promote elevated transendothelial leukocyte infiltration and accelerate synovial inflammation together with the destruction of bone and cartilage destruction. In contrast, the suppression of joint neovascularization can alleviate synovitis and pannus formation [6]. This study revealed that MVD and the scores for pannus formation in CIA mice were reduced in a dose-dependent manner in response to PTX treatment. These findings suggest that PTX is effective for inhibiting angiogenesis. In addition, the correlation analysis indicated that MVD was positively correlated with pathological scores in CIA mice, including synovitis scores, pannus formation scores, and bone destruction scores. These results may support an ability of angiogenesis to stimulate inflammation and bone destruction; the suppression of joint neovascularization can also alleviate synovitis and pannus formation. These findings offer new insights into the treatment of RA. In future studies, we aim to assess whether PTX can suppress angiogenesis in RA.

VEGF is a type of growth factor that is secreted by macrophages and synovial tissue fibroblasts in RA [39], and it is a key regulator of angiogenesis. VEGF can promote the proliferation and migration of endothelial cells to support the emergence of new blood vessels. It also plays a direct pro-inflammatory role in the pathogenesis of RA [40]. Numerous studies have validated that high levels of VEGF are present in the serum, synovial tissue, and fluids of patients with RA [41]. In addition, the levels of VEGF are positively correlated to the disease activity scores and erythrocyte sedimentation rate (ESR) [42]. In the CIA mouse model, preventative treatment of anti-VEGF antibody delayed CIA onset, vascularization, and swelling. In contrast, post-onset treatment with anti-VEGF antibodies did not affect the development or severity of arthritis [43], indicating that angiogenesis regulated by VEGF plays a critical role in the early stage of arthritis progression.

Hypoxia is induced by the metabolic demand of the increasing number of leukocytes recruited into the RA joints, which can lead to the accumulation of HIF-1α in the cytoplasm, and induces the expression and secretion of VEGF by macrophages as well as RA synovial tissue fibroblasts [8, 44]. Furthermore, positive feedback regulation of the HIF-1α and VEGF pathways can trigger angiogenesis during hypoxia [45]. In vitro studies have confirmed that the absence of HIF-1α attenuates angiogenesis through a VEGF-dependent mechanism [46]. In contrast, conditional knockout of HIF-1α in myeloid cells can relieve experimental arthritis by reducing myeloid cell activation independently of VEGF, indicating that HIF-1α can also modulate the alleviation of arthritis and inflammation independently of its anti-angiogenesis effect [47]. This study indicated that PTX can significantly decrease the expression of VEGF and HIF-1α in synovial tissues and peripheral serum of CIA mice. The inhibitory effect is dose-dependent. Additionally, the levels of VEGF and HIF-1α in the synovium were positively correlated to MVD. We posit that VEGF and HIF-1α may be the targets through which PTX inhibits the formation of microvessels in CIA mice, making them potential therapeutic targets for RA.

This research has some limitations. First, PTX has anti-angiogenic effects in mice, but this phenomenon has not been examined in vitro such as in FLSs. Second, although the expression of VEGF and HIF-1α was alleviated after PTX treatment, the upstream signaling pathways and other mechanism must be clarified in future studies. Furthermore, we did not evaluate the safety profile of PTX in this study. Therefore, further studies are necessary to explore the pathophysiologic role and safety of PTX in RA.

## Conclusions

In conclusion, this work suggests that PTX may alleviate CIA by suppressing angiogenesis, providing new insights into the treatment of RA. VEGF and HIF-1α may be the targets through which PTX suppresses the formation of microvessels.

CFA, complete Freund’s adjuvant; CIA, collagen-induced arthritis; CII, collagen II; DCs, dendritic cells; ESR, erythrocyte sedimentation rate; FLSs, fibroblast-like synovial cells; HIF-1α, hypoxia-inducible factor-α; IOD, integrated optical density; MDSCs, myeloid-derived suppressor cells; MMPs, matrix metalloproteinases; MVD, microvessel density; PTX, taxol; RA, rheumatoid arthritis; TNF-α, tumor necrosis factor α; VEGF, vascular endothelial growth factor
